# The role of physique, strength and endurance in the achievements of elite climbers

**DOI:** 10.1371/journal.pone.0182026

**Published:** 2017-08-03

**Authors:** Mariusz Ozimek, Robert Rokowski, Paweł Draga, Vladimir Ljakh, Tadeusz Ambroży, Marcin Krawczyk, Tomasz Ręgwelski, Arkadiusz Stanula, Karol Görner, Adam Jurczak, Dariusz Mucha

**Affiliations:** 1 Department of Track and Field Sports, Institute of Sport, University School of Physical Education, Cracow, Poland; 2 Department of Alpinism and Tourism, Faculty of Tourism and Recreation, University of Physical Education, Cracow, Poland; 3 Department of Physical and Sport Education, University of Warsaw, Warsaw, Poland; 4 Department of Sports Theory and Anthropometric, Institute of Sport, University School of Physical Education, Cracow, Poland; 5 Department of Gymnastics, Institute of Sport, University School of Physical Education, Cracow, Poland; 6 Doctor Courses, Institute of Sport, University School of Physical Education, Cracow, Poland; 7 Department of Individual Sports, The Jerzy Kukuczka Academy of Physical Education, Katowice, Poland; 8 Filozoficka Faculta, Department of Physical Education and Sports, Matej Bel University, Banská Bystrica, Slovakia; 9 Department of Theory and The Methodology of Physical Education, University School of Physical Education, Cracow, Poland; 10 Department of the Biological Renovation and Correction of Defects of Attitudes, Institute of Biomedicine, University School of Physical Education, Cracow, Poland; Texas A&M University, UNITED STATES

## Abstract

**Purpose:**

The primary aim of this study is to determine the principal somatic and motor determinants for elite climbers.

**Methods:**

Twenty climbers were examined [age: 28.5±6.1 years].The runners were divided into two groups based on their climbing level, according to the International Rock Climbing Research Association (IRCRA). Elite climbers represented a 8b-8c Rotpunkt (RP) climbing level (n = 6), and advanced climbers represented an 7c+-8a RP level (n = 14). The following measurements were assessed: height, weight, lean body mass, upper limb length, arm span, and forearm, arm, thigh and calf circumference. The BMI, Rohrer ratio, and Ape Index were also measured. The following motor tests were assessed: a specific test for finger strength, an arm strength test, and a test of muscle endurance (hanging from 2.5 and 4 cm ledges). In addition, pull ups were used to measure muscle resistance to fatigue.

**Results:**

Elite climbers recorded significantly higher values for finger strength than advanced climbers (129.08 vs. 111.54 kg; t_(18)_ = 2.35, p = 0.03) and arm endurance (33.17 vs. 25.75 pull ups; t_(18)_ = 2.54, p = 0.02). In addition, the calf circumference was significantly lower in elite climbers than that in advanced climbers (34.75 vs. 36.93 cm; t_(18)_ = 3.50, p = 0.003).

**Conclusion:**

The results suggest that elite climbers have greater finger strength and arm endurance than advanced climbers.

## Introduction

Sports disciplines are typically classified based on the type of motor activity involved, including endurance (long-term activities), forceful-high speed (activities with high intensity and short duration) and mixed disciplines, in which the activity is categorized as either forceful-endurance or high speed-endurance.

Sport climbing is considered a forceful-endurance discipline in which the main resistance force is the body weight. The effort exerted during this activity has an intermittent nature, which is characterized by numerous isomeric muscle contractions. The duration of climbing on rocks and artificial walls varies from a few to several minutes. As in other sport disciplines, the type of exertion determines the physique and the indicators that characterize the climber’s morphological physique [1–3].

Watts et al. [4] observed that elite climbers were characterized by a lower weight and a lower fat composition than non-climbing individuals. Similar results were noted by Rokowski and Tokarz [5]. Elite climbers can be distinguished from recreational climbers by their lower weight and lower fat composition. Notably, comparisons between elite climbers and non-climbers or recreational climbers only enable us to identify the essential determinants of sports results. It is difficult to judge which somatic features are important to climbing at the elite advance 8b-8c Rotpunkt (RP) level. To gain a greater understanding of this question, it is necessary to compare the physique of professional performers to that of advanced climbers and calculate the linear correlation coefficients between rock climbing levels and somatic features. In a study performed by Espana-Romero et al. [6], no essential differences in physique were observed between elite (8b on-sight) and expert (8a on-sight) climbers. Thus, the presented study results demonstrate that although elite climbers are characterized by a specific physique, there is no difference between these elite climbers and climbers representing the 8a on-sight level.

In publications dedicated to sport climbing, studies have placed importance on the relative strength of the upper limbs [4–7]. Studies related to strength in sport climbing have been conducted using dynamometers and specific tests. In the observations made by Watts et al. [4], Grant et al. [8], and Rokowski and Tokarz [5], the best results were consistently achieved by elite climbers when compared to recreational climbers, and these tests were related to the strength of both the fingers and arms [5]. These results indicated the significant role of finger and arm strength in sport climbing. Nevertheless, based on these studies, it was difficult to judge the importance of finger and arm strength for achieving the 8c RP climbing level. Ozimek et al. [9] attempted to solve this problem in their study, which demonstrated that finger strength in elite climbers correlates significantly with climbing level; however, no correlation was observed for arm strength. The above study results suggest that in terms of strength abilities, finger strength is important in differentiating professional climbers and enables climbers to achieve the 8c RP climbing level.

In sport climbing, the length of exertion varies from a few to several minutes. Thus, it is possible to state that in addition to strength, a high level of endurance is also important. This hypothesis has been confirmed by numerous scientific reports. In a study performed by Ferguson et al. [10], more advanced climbers achieved better results in tests related to maintaining 40% of the dynamometric clamping force. The test consisted of 5 seconds of tension followed by 2 seconds of forearm muscle relaxation. Another study conducted a dynamometer by Vigouroux et al. [11]. The climbers were placed in a sitting position and performed intermittent exertion, which involved clenching the dynamometer using a certain fingertip rhythm. The researchers observed that professional climbers maintained 80% of maximal strength for a longer period of time. In addition, those authors noted that climbers achieved functional equilibrium at 60% strength maximum while the control group achieved functional equilibrium at 50% strength maximum.

In addition, Ozimek et al. [9] (study conducted on a group of 15 climbers with rock climbing levels between 8c and 8a RP) reported a significant correlation coefficient between the rock climbing level and the results of the ledge hanging test with widths of 2.5 and 4 cm. Similar results were reported by Balaś et al. [12]. These results indicate that endurance, more precisely, muscle resistance against fatigue during isometric contraction, is a very important factor in sport climbing. Moreover, it is possible to conclude that together with strength, these factors are the priority components that enable climbing at grade 8c RP. The cited studies require further proof; therefore, the primary aim of this study is to determine the primary somatic and motor determinants for elite climbers. We put forward a claim that the highest-tier climbers present similar somatic features to those of the lower tier, yet the former are superior to the latter in selected special fitness tests.

## Methods

Twenty male climbers were included in this study [age: 28.5±6.1 years]. The participants were placed into the following two groups based on their climbing level, according to the International Rock Climbing Research Association (IRCRA): elite climbers at Rotpunkt (RP) level 8b-8c (n = 6) and advanced climbers at RP level 7c+-8a (n = 14). This study was approved by the BioEthics Committee (No. 42/KBL/OIL/2015) (S1 Fig). The following parameters were measured: height, weight, lean body mass, length of the upper limb, arm span, and the circumference of the largest forearm, arm, thigh and calf. In addition, the BMI, Rohrer ratio, and Ape Index were measured.

### Anthropometrics

Body height was measured from the bottom of the feet to the vertex of the head while standing erect. For the arm span measurement, subjects were standing in an upright orthostatic position with arms and fingers fully extended in lateral abduction at a 90° angle with the trunk. The particular body-segment length measurements were completed using appropriate anthropometric instruments (Sieber Hegner Maschinen AG, Switzerland). Climber’s lean body mass was calculated following the body fat assessment. Body fat percentage was determined from the estimate of body density. The Durnin & Wormersley [13] body density equations with measurement of 4 skinfolds (biceps, triceps, subscapular, suprailiac) results were used. The results obtained from the equations (that of body fat density) were subsequently taken into the Siri equation to calculate the body fat [14]. Constant spring pressure of (10 g·mm^-2^) Harpenden Skinfold Calliper (Holtain Ltd., UK.) was used for measurement of skinfold thickness. The anthropometry measurements were conducted with the reliability result of–ICC_(3,1)_: 0.997; 95% lower; upper CI: 0.996 and 0.998, respectively.

### Finger and arm strength measures

**Fig 1:**
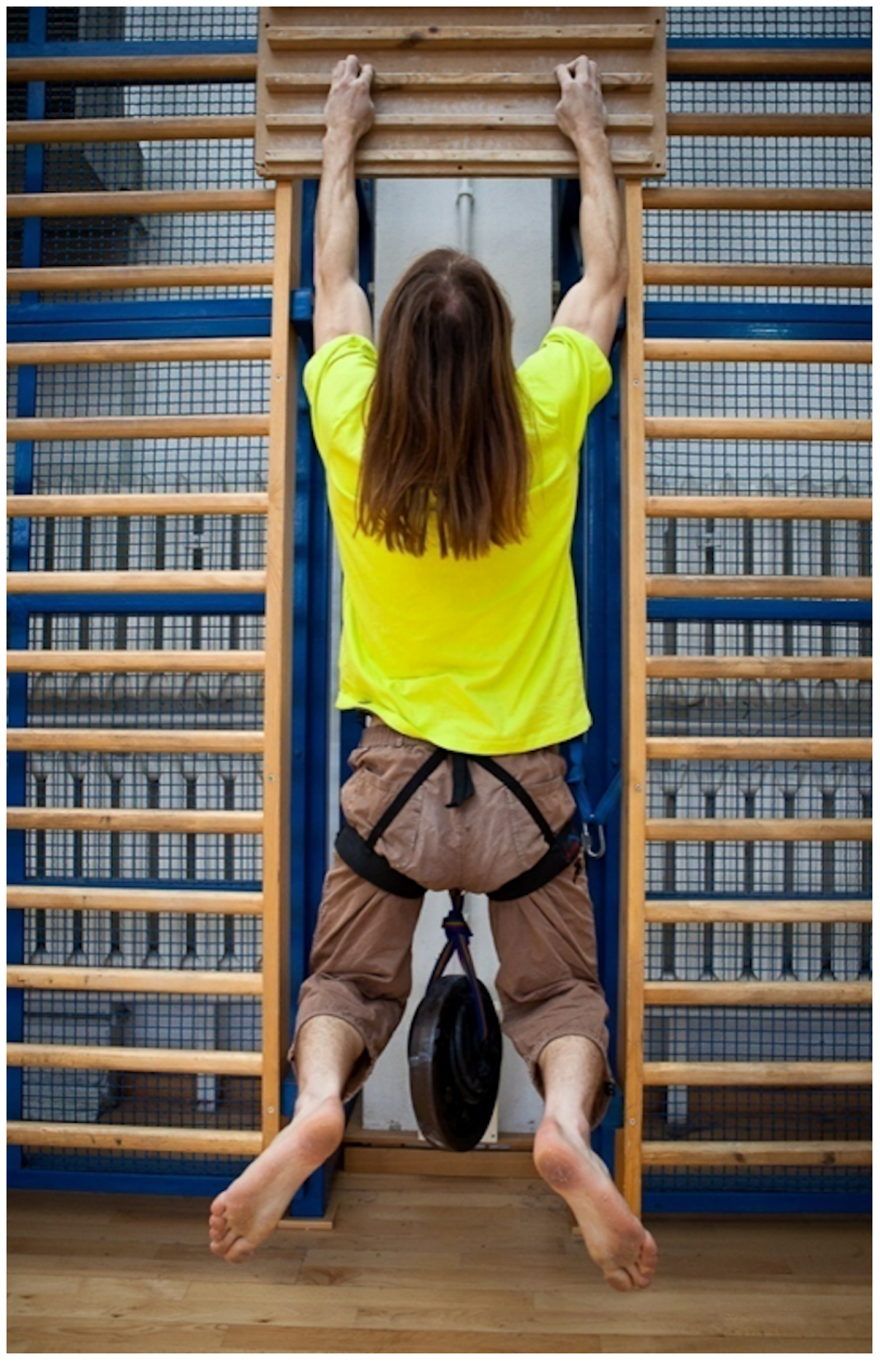
Finger strength test.

In this study, the subjects were asked to grip a ledge with a width of 2.5 cm and to hang vertically with the maximum weight they could still lift attached to a hip belt (see Fig 1). The grip was performed with only four fingers of each hand (without the thumbs) and with the hands at shoulders’ width apart. The subjects were required to maintain this position for 3 seconds. The results of this test were recorded as the absolute values of the sum of the body mass and the additional weight [kg] and as the relative strength (i.e., without the body mass). In the next sections, those results are termed LEDGE 1 and LEDGE 2, respectively.

**Fig 2:**
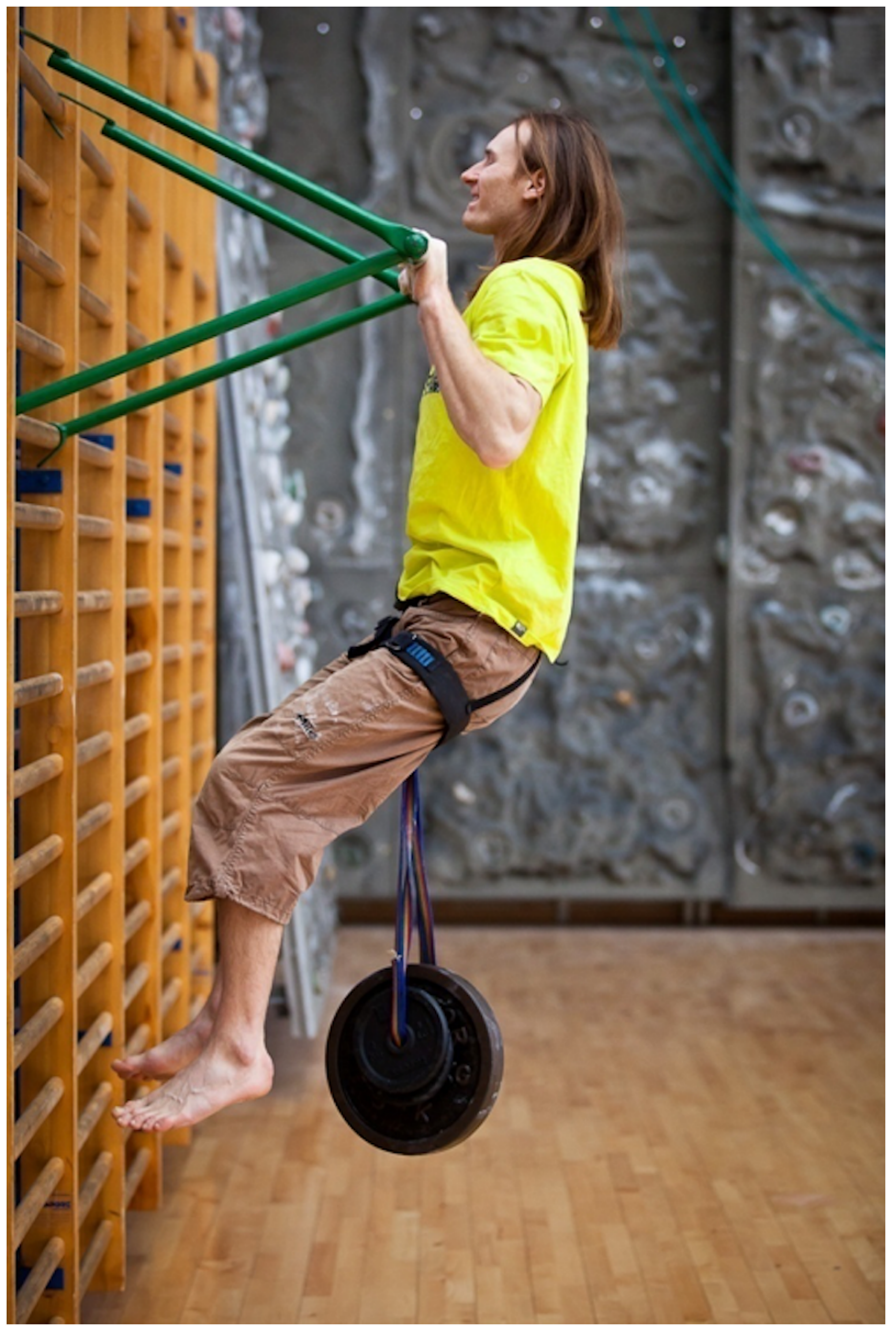
Arm strength test.

The measurement of the climbers’ strength was combined with a test to assess the strength of their arm muscles (see Fig 2). The subjects were asked to do a pull up, bringing the chin over the bar with the maximum weight they could lift attached to a harness. An overhand grip was required, and the hands were to be held at shoulders’ width. The results of this test were recorded (with an accuracy of 1 kg) as both absolute and relative values. For the purpose of further analyses, these results are denoted by BAR 1 and BAR 2, respectively.

### Specific motor tests for endurance measurements

The group of tests that assessed the climbers’ muscle endurance consisted of specific motor tests.

Endurance (the resistance of muscles to fatigue) was examined using specific motor tests. The first test (HANG 1) required the subjects to hang from a ledge with a width of 2.5 cm. The second test (HANG 2) was only different in that a 4 cm ledge was used. In both tests, the subjects were required to grip the ledge with four fingers of each hand (the thumb was left disengaged), with the hands held at shoulders’ width, with the upper extremities straight, and with the body hanging vertically as long as possible (until failure). The outcome of these tests was the length of time the subject could maintain this position, which was measured with an accuracy of 1 s.

Pull-ups were used to measure muscle resistance to fatigue. In this test (PULL UPS), the maximum number of pull ups performed by the subject was recorded; however, for a pull up to be credited, it had to be performed in accordance with the IPFT rules.

### Statistical analysis

Basic descriptive statistics were computed for the somatic and motor tests in each group of climbers. Differences between the groups were assessed using the Student’s t-test or the Mann-Whitney test. Spearman’s rank correlation coefficients were calculated between the rock climbing level and the somatic types and the motor tests studied. Data are presented as mean, standard deviation (SD) and mean difference between groups and 95% confidence intervals as appropriate. The computations were performed using the STATISTICA 12 PL software for Windows on University School of Physical Education, Cracow, Poland.

## Results

Table 1 depicts the climber’s somatic and motor characteristics and the differences between groups. The results showed that elite climbers had an average height, a lean body mass, and low values for weight, fat composition, body mass index and Rohrer’s indicator. In addition, elite climbers had a smaller thigh and calf circumference than non-climbing individuals. Additionally, elite climbers vary in average arm and forearm circumference relative to the population norm [15]. Furthermore, arm span, ape index, and length of the upper limb did not differ significantly between elite climbers and non-climbing individuals. The main target of the study was not to describe the somatic and motor characteristics of elite climbers but to establish the primary somatic and motor determinants of climbers that determine their ability to achieve a climbing grade level of 8b-8c RP.

**Table 1:** Somatic characteristics of the examined climbers and intergroup differences.

Variables	Elite (n = 6)			Advanced (n = 14)			Elite minus Advanced
	Mean	SD	CV	Mean	SD	CV	Mean difference (95% CI)
Height [cm]	177,38	5,61	3,16	177,85	3,74	2,10	–0,47 (–4,92; 3,98)
Weight [kg]	66,92	5,83	8,70	71,11	4,28	6,02	–4,2 (–9,08; 0,69)
Lean body mass [kg]	58,93	4,00	6,70	61,54	2,97	4,82	–2,61 (–5,98; 0,76)
Fat mass [%]	7,97	4,83	60,60	14,29	22,79	159,45	–6,33 (–26,35; 13,7)
BMI	21,18	1,04	4,90	22,25	1,46	6,56	–1,07 (–2,46; 0,32)
Rohrer’s ratio	1,20	0,06	5,24	1,27	0,09	7,49	–0,07 (–0,16; 0,02)
Arm span [cm]	180,00	5,43	3,00	181,87	5,61	3,08	–1,87 (–7,57; 3,83)
Ape index [cm/cm]	1,01	0,02	1,60	1,02	0,02	1,77	–0,01 (–0,03; 0,01)
Length of the upper limb [cm]	76,82	2,61	3,40	77,96	2,15	2,76	–1,15 (–3,49; 1,2)
Arm circumference [cm]	28,92	1,36	4,60	30,21	2,05	6,80	–1,3 (–3,23; 0,64)
Forearm circumference [cm]	28,50	1,05	3,60	28,63	1,11	3,89	–0,13 (–1,25; 0,99)
Thigh circumference [cm]	50,92	2,91	5,70	53,09	2,37	4,47	–2,17 (–4,76; 0,42)
Calf circumference [cm]	34,75	0,82	2,30	36,93	1,41	3,82	–2,18† (–3,49; –0,87)

SD–standard deviation; CV–coefficient of variance [%]. Statistically significant differences between the elite and advanced groups at:

^†^ p<0.01.

Valuable information related to the differences between elite and advanced climbing competitors was obtained in this study. The conducted experiments demonstrate that the factor that differentiates elite climbers is a smaller calf circumference, as shown by the existence of a statistically significant difference of 2.2 cm in favor of the advanced climbers. It is worth mentioning that the calf circumference was also smaller in the group of elite climbers. The difference was 2.1 cm; however, this result was not statistically significant. Additional data regarding the body components associated with achieving a climbing level of 8c-8b RP are provided by Spearman correlation rank analysis. A significant correlation coefficient was only observed for the correlation between the RP level and calf circumference (R = –0.53). This study confirms previous findings that elite climbers have a higher strength level and increased endurance than that of recreational climbers [2,8]. Our findings demonstrate that finger strength and stronger force endurance in the arms differentiate elite climbers (Table 2). In addition, the results indicate that resistance of the forearms to fatigue is essential for elite level climbers.

**Table 2:** Differences in motor characteristics between the examined groups of climbers and intergroup statistics.

Variables	Elite (n = 6)			Advanced (n = 14)			Elite minus Advanced
	Mean	SD	CV	Mean	SD	CV	Mean difference (95% CI)
Ledge 1 [kg]	129,08	15,01	11,63	111,54	15,36	13,77	17,54* (1,89; 33,19)
Ledge 2 [kg/kg]	1,93	0,20	10,32	1,58	0,26	16,78	0,35† (0,1; 0,61)
Bar 1 [kg]	121,10	11,13	9,19	121,83	13,02	10,69	–0,73 (–13,57; 12,11)
Bar 2 [kg/kg]	1,80	0,04	2,29	1,71	0,17	10,07	0,09 (–0,06; 0,25)
Hang 2.5 cm [s]	74,67	18,96	25,39	53,66	13,51	25,18	21,01* (4,2; 37,82)
Hang 4 cm [s]	100,00	23,71	23,71	80,42	7,09	8,82	19,58* (3,51; 35,66)
Pull Ups [n]	33,17	7,25	21,86	25,75	5,42	21,04	7,41* (1,28; 13,55)

SD–standard deviation; CV–coefficient of variance [%]. Statistically significant differences between the elite and advanced groups at:

*p<0.05,

^†^p<0.01.

We also examined the correlation coefficients between the rock climbing level and the test results for the HANG 1 and HANG 2 test (Table 3). The correlation coefficients also confirm the importance of relative finger strength (R = 0.52).

**Table 3:** Spearman rank correlations between RP level and the examined somatic features and motor test results in elite and advanced climbers.

Variables	R—Spearman
Height [cm]	–0.17
Weight [kg]	–0.43
Lean body mass [kg]	–0.41
FT [%]	–0.41
BMI	–0.31
Rohrer indicator	–0.23
Arm spam [cm]	–0.33
Ape index [cm/cm]	–0.38
Lenght of the upper limb [cm]	–0.36
Arm circuits [cm]	–0.32
Forearm circuits [cm]	0.08
Thigh circuit [cm]	–0.28
Calf circuit [cm]	–0.53*
Ledge [kg]	0.4
Ledge relative [kg/kg]	0.52*
Bar [kg]	–0.31
Bar relative [kg/kg]	0.15
Hang 1 [s]	0.45*
Hang 2 [s]	0.5*
Pull lups [n]	0.36

Statistically significant differences between the elite and advanced groups at:

*p<0.05.

## Discussion

To date, research has indicated that elite climbers are characterized by an average height. These climbers are also characterized by a low weight, a low body fat composition and a low body mass index when compared to non–climbing individuals or recreational climbers [1,3,8]. It is worth noting that the aim of this study was to identify the important somatic and motor determinants for climbers who achieve the 8b-8c RP level. Therefore, a comparison of elite and advanced climbers was conducted, and the correlation coefficients between the rock climbing level and the examined somatic features and motor tests were assessed. The results indicate that elite climbers can be distinguished from advanced climbers based on their smaller calf circumference, with a difference of up to 2.2 cm. This difference was the only statistically significant difference identified in this study. Although the differences in the other parameters were not statistically significant, elite climbers also had a lower weight than advanced climbers, with a difference of up to 4.2 kg, and a lower fat composition of approximately 6.3%. In addition, elite climbers were characterized by a lower lean body mass than advanced climbers, with a difference of 2.6 kg. Additionally, the examinations demonstrated that elite climbers were characterized by a significantly lower Rohrer indicator than advanced climbers. This finding indicates that elite climbers have a more slender build (Table 1) The above data partially corresponds with the results reported by Espana-Romero et al. [6] and prove that the somatic requirements of this discipline are a low weight, a low fat composition, and a slender build. Nevertheless, based on the tests conducted in this study, the primary factor that distinguishes elite climbers is a smaller calf circumference. However, according to us, we should carefully refer to the conclusions, which show the importance of low circuit of low calf in elite climbing group achievements. We would like our readers to notice, that our exam group consisted of performers who compete at a high rock climbing level and which followed the natural selection. According to our study low circuits of low calf as well as thigh (the difference is not statistically significant but distinct) can be related to the low weight of the elite climbers. Whereas, low body mass distinctly conditions one of the most essential climbing abilities, namely the finger’s strength levels [1,12]. At this point, we would like to add that in the elite climbers group, there were people somewhat divergent from the average of this group in terms of low calf circuit or tight Table 1 This phenomenon can be explained by feature compensation. It is related to the fact that a competitor with structural deficiencies can compensate for these deficiencies with other important factors such as technique or coordination potential [16]. In addition, it wonders the lack of intergroup differences in self-examination in the case of arm and forearm circuits. Probably, it results from the importance in climbing of both strength and endurance of upper limbs. The point is that too much muscle mass of the arm and forearm could worsen the strength indicators of the above structures. This data, however, does not correspond to the results of the Espana-Romero [6] study where significant correlation coefficients of the forearm circuit with the rock’s score of r = 0.8 were noted.

In addition, this study found that sport climbers can be characterized by higher relative finger and arm strength and increased resistance to fatigue in the forearms compared to non-climbers or recreational climbers [5, 10–12]. The results obtained from this study indicate that elite climbers can be distinguished from advanced climbers based on high levels of finger strength and arm strength endurance. This finding is evidenced by intergroup differences in favor of elite climbers (Table 1).

The significant influence of finger strength–in relative terms–on the effectiveness of artificial wall climbing is a result of the specific requirements of this sports discipline, as climbers must often have to hang by their fingertips against the force of gravity. The difficulty of the climbing route is strictly linked to the size of the holds. Therefore, finger strength, especially in relative terms, is an important factor that influences rock climbing achievements among elite climbers.

However, the explanation for the lack of intergroup differences relating to arm strength remains unclear. This phenomenon can be explained by the fact that while climbing, there are no situations in which the competitor must pull up on one hand to reach the next hold. Even in horizontal formations (i.e., roofs), the climber is standing up or hooks the legs (with the heel or toes depending on the situation), using the slightest uneven ground and therefore crossing the upper limbs. Thus, the current study appears to be correct in identifying finger strength and not arm strength as the principal determinant that enables climbing on 8c-8b RP routes. Notably, these results are consistent with previously reported results [9].

Climbing usually takes a few to several minutes. During this period of exertion, there is a significant amount of isometric muscle tension [17]. Thus, in different scientific exams, a significant correlation coefficient was observed between the rock climbing level and the results of the ledge hanging test with widths of 2.5 and 4 cm [9,12]. The results of own studies are not evident in these regards. On the one hand, the statistically significant differences between the examined groups have not been noted (although it should be noted that the absolute differences were in favour of the elite climbers and amounted to 11,1–15 sec.). On the other hand the significant correlation coefficients between RP level at the rocks and the ledge width 2,5 cm and 4 cm hanging time have been noted (R = 0,45–0,5).

This data indicates the importance of arm strength endurance for elite climbers. This result is not surprising because the requirements of sport climbing necessitate the constant bending and strengthening of the arms. Therefore, maintaining a certain level of arm strength is likely an important element that enables climbing at the 8b-8c RP level.

## Conclusions

Elite climbers should have a low weight, a low fat composition, and a slender body build. Elite climbers can also be distinguished from advanced climbers based on finger strength and arm isometric endurance.

## Supporting information

S1 Fig BioEthics Committee (No. 42/KBL/OIL/2015). (JPG)
