# Supplementary figures and images for: The role of physique, strength and endurance in the achievements of elite climbers
Source: PLoS One. 2017 Aug 3;12(8):e0182026. doi: 10.1371/journal.pone.0182026 (PMC5542533; doi:10.1371/journal.pone.0182026)

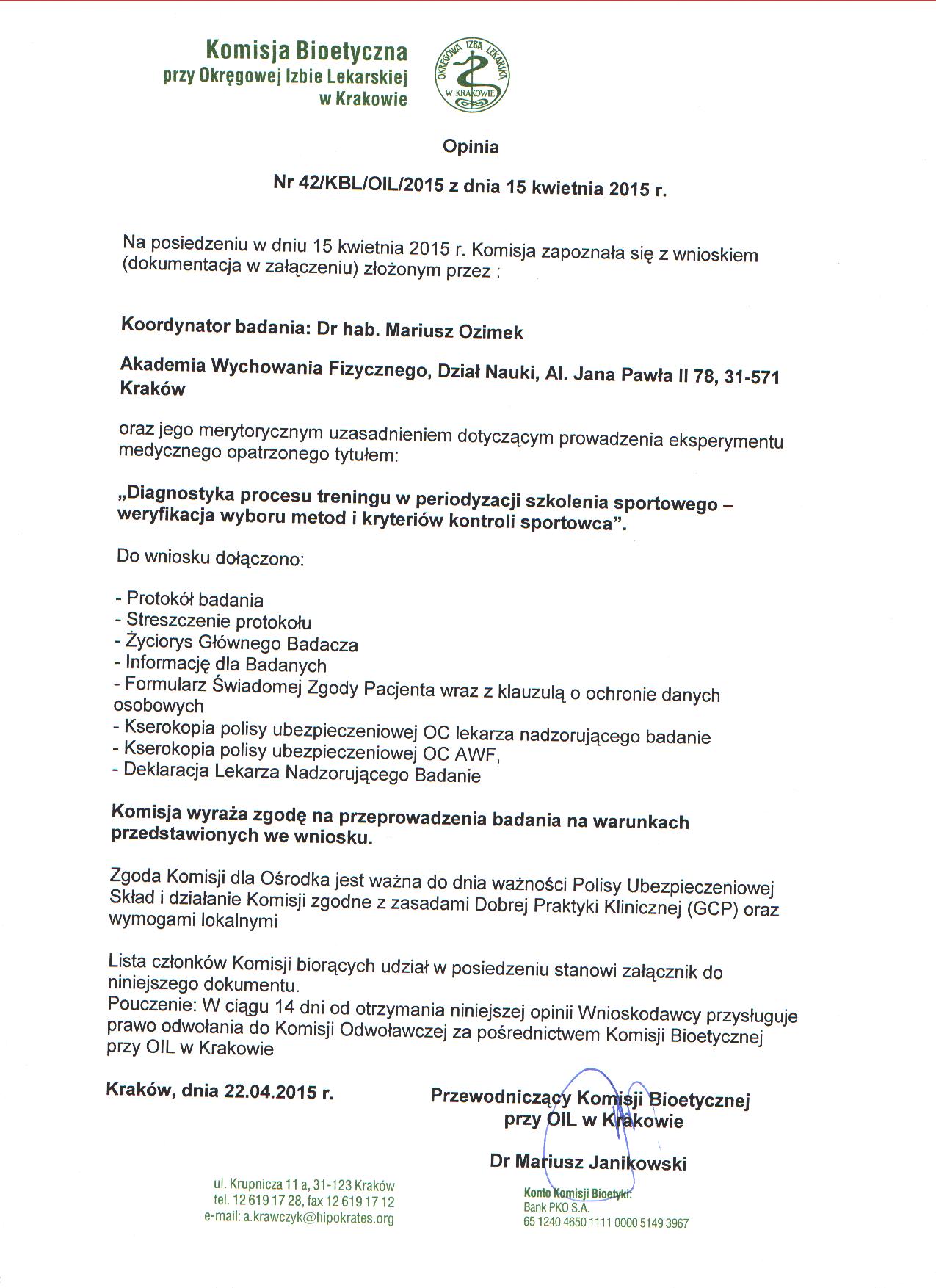

Supplement: S1 Fig — (JPG) [file pone.0182026.s001.jpg]
